# Frontrunning, Free-Riding and Over-Aspiring: A Case Study Exploring How Configurations of Involvement, Social Comparison and Organizational Goal Attainment Affect Perceived Network Goal Attainment

**DOI:** 10.34172/ijhpm.8050

**Published:** 2024-08-18

**Authors:** Galina van der Weert, Katarzyna Burzynska, Joris Knoben

**Affiliations:** ^1^Talma Institute, Vrije Universiteit, Amsterdam, The Netherlands; ^2^Institute for Management Research, Radboud University, Nijmegen, The Netherlands; ^3^Tilburg School of Economics and Management, Tilburg University, Tilburg, The Netherlands

**Keywords:** Primary Care Networks, Involvement, Social Comparison, Organizational Goal Attainment, Network Goal Attainment

## Abstract

**Background:**

Complex problems in healthcare (fragmentation, specialization, and increased costs) are often addressed by implementing collaborative interorganizational networks. Theoretical models prescribe organizational practices that should ensure the effectiveness of these networks. However, these models are mostly aimed at organizing networks to achieve optimal effectiveness. One of the mechanisms increasing effectiveness, is the involvement of network members. We argue that even though network involvement may be high, there are mechanisms at play that decrease the member’s perceived network goal attainment, resulting in dissatisfied and dissociated members. One of these mechanisms is the comparison of input and output versus the input and output of other members; while the other is the pursuit of organizational goals by network membership. In combination with each other, these may lead to low perceived network goal attainment.

**Methods:**

We apply a mixed method study in a local primary care network (PCN) in the Netherlands. We collect and analyse two types of data: (1) interviews, analysed using thematic analysis, and (2) surveys, analysed using crisp-set qualitative comparative analysis (QCA).

**Results:**

We found three different pathways to low perceived network goal attainment. Members that are highly involved with the network, can still feel dissatisfied with the network’s goal attainment if they engage in social comparison or if they pursue organizational goals rather than network goals by network membership. We called these pathways over-achieving, and frontrunning. The third pathway, freeriding, describes members that are not very much involved in the network, but pursue organizational goals rather than network goals, and are also dissatisfied about the network’s goal attainment.

**Conclusion:**

Network member involvement positively affects perceived network goal attainment. We argue however, that even high network involvement can result in low perceived network goal attainment. Member’s comparison of each other’s input and output, as well as the pursuit of organizational goals, result in low perceived goal attainment even if members’ involvement is high. Future research aimed at network level effectiveness should take member level characteristics and sociodynamic factors into account.

## Background

**Key Messages**

**Implications for policy makers**

Currently the implementation of collaborative networks is a common policy solution to the complex problems the healthcare sector faces. These networks are organized to obtain optimal effectiveness. Collaborative networks are not a bad policy instrument per se, but we argue that it is important to consider the following:

- Organizational members in primary care networks (PCNs) are often representatives of their own companies, who rely on a steady inflow of patients. This means that all aspects of human social behaviour, such as social comparison, are at play in these networks. It is therefore not enough to only consider organizational measures and technical network descriptions.
- Research about interorganizational networks in healthcare should be designed to also consider network member level characteristics and sociodynamic aspects that could affect network effectiveness, such as social comparison.
- The pursuit of organizational goals affects perceived network goal attainment, even if network members are highly involved. Therefore, pro-competitive policy, such as health insurance marketization, affects value proposition of network membership differently, for different healthcare providers. Moreover, financial rules and regulations affect the value proposition of members for participating in a (primary) care network. This, in turn, affects the effectiveness of the network.

**Implications for the public**

Primary care networks (PCNs) are increasingly salient in the Dutch healthcare sector. General practitioners (GPs) collaborate with different (primary) care providers in healthcare centres to improve accessibility and quality of care, while reducing costs. These networks do not always function effectively. This can be explained by feelings of inequity through a process of social comparison, by members who feel involved with the network. Additionally, the pursuit of organizational goals over network goals may affect the perception of network goal attainment negatively. Even though PCNs have previously shown positive effects for citizens and patients, such as improved continuity of care, there may be other aspects on the organizational side of the network at play that affect the effectiveness of the network.

In healthcare, the implementation of interorganizational networks is related to a wider implementation of value based healthcare^1^ and integrated care.^2^ Specifically in primary and chronic care it is necessary to improve continuity of care and arrange shared responsibility for care paths and population health.^2^ As people grow older they are burdened with more chronic diseases and conditions. Simultaneously, medical innovations allow for more complex treatments. The result is fragmentation and increasingly complex healthcare delivery against ever increasing costs.^3^ In interorganizational networks in healthcare, multiple care providers collaborate in varying constellations to solve these problems.

 An interorganizational network in healthcare builds connections and relationships between different care providers in order to achieve continuity of care or reduce care fragmentation.^2^ The definition of an interorganizational network in organization sciences is as follows: a constellation of at least three organizations that work together because they aim towards a common goal.^4^ Moreover, these organizations are autonomous and there is no hierarchical system between them. Member organizations expect that every other member invests in achieving a commonly defined goal. Their mutual contribution, as well as the previously defined common goal, build a shared identity and simultaneously bounds the network.^5^ Many organizations in healthcare are currently part of multiple interorganizational networks.^6^ Importantly, this non-hierarchical interdependency between organizations requires a form of coordination and structuring. Activities need to be aligned, resources allocated and conflicts prevented or mitigated.^7,8^ There is substantial knowledge on how to govern these interorganizational networks, but still we find that a lot of the initiatives are unsuccessful or unsustainable.^9,10^ Even though this is a widely acknowledged and important topic, empirical studies on network effectiveness are fragmented and few in number.^11-13^

 Many models try to capture the complex constellation of factors explaining network effectiveness in general, but also more specifically in healthcare.^7,14-16^ Most of these models entail technical factors such as structure, governance, and coordination tools^17^ – all with the assumption that the network will be successful with the “right” constellation of these factors. These theoretical models provide important insights into why networks (fail to) obtain their goals, but there are still large knowledge gaps in how informal or intangible factors affect network effectiveness.^14^ Therefore, this study aims to shed light on the role of such factors in shaping network effectiveness.

 “*I can collaborate quite well with all the organizations in the network. But once they stop putting in effort but still gain benefits, I get angry, because that is just unfair towards my people, who spend considerable time in this collaboration” *(Hospital chief executive officer [CEO], member of several interorganizational networks; personal communication, November 2019).

 The statement of this CEO sparked our interest in the mechanisms that are at play in interdependent relationships between organizations in collaborative networks. The CEO acknowledges the fact that it is difficult to keep all the parties involved equally throughout the existence of the network; but at the same time, without being explicit, mentions that he keeps track of how other member organizations behave within the network. The CEO also suggests that the amount of hours invested by his employees matters as they are capital investments. In short, the CEO states that his involvement and his satisfaction with the network are related to his perception of other member’s efforts as well as how the efforts of his organization, such as the hours his employees put in, relate to his organizational outcomes.

 In this article we contribute to the conceptualization of the effectiveness of interorganizational healthcare networks at the organizational level. First we explain how configurations of involvement, social comparison and organizational goal attainment can possibly contribute to network members’ perceived goal attainment. Second, we study these configurations in a primary care network (PCN) in the Netherlands that consists of almost thirty organizational care delivery practice members. Third, with qualitative comparative analysis (QCA) we describe pathways or configurations that lead to lower perceived network goal attainment. This can explain why initially highly involved network members may ultimately become dissatisfied with the network’s achievements.

###  Theoretical Background: Factors Affecting Perceived Network Goal Attainment in Interorganizational Networks in Healthcare

 Organizing networks between organizations stems from the belief that collaboration will achieve more than the sum of its parts. The importance of “soft” factors for network effectiveness is previously emphasized,^17^ but still “soft” factors are often measured at a network *or* actor level (information sharing, communication, culture and trust), while insight in the mechanisms of how such factors affect network goal attainment, is limited.

####  Network Goal Attainment 

 As networks become more prevalent, so does the need to take them seriously – and along with taking networks seriously, comes the necessity to evaluate their effectiveness.^18^ The effectiveness of a network can be conceptualized in myriad ways.^16,19-21^ Not only because network effectiveness is a multifaceted construct,^18^ but also because network members may perceive the definition of network effectiveness differently.^21^ Most often network effectiveness is defined at the participant organization or network member level, the network level and ultimately the community level.^16^

 Network effectiveness at the network level is defined as “the attainment of positive network-level outcomes that could not normally be achieved by individual, organizational participants acting independently.”^7^ At the community level: “networks should be judged based on the contribution they make to the community they are trying to serve.”^16^ The reason to assess network effectiveness at the participant/organization level, is the fact that most organizations participate in network collaboration out of self-interest: they believe that, by contributing to a common goal, in the process, their own organizational goals will be more easily achieved as well. Moreover, when individual network members do well, their contribution to achieving the common goal is enhanced.^16^ Hence, very often, network effectiveness for the individual organizational network member is defined in terms of legitimacy of the organization in the community, resource acquisition, client outcomes and costs.^16^

 Several literature reviews have provided an overview of network effectiveness measures.^5,19-21^ The conclusion of these reviews is that there are almost as many ways to conceptualize network effectiveness as there are studies about effectiveness. However, there are two methods that are often applied. The first is quantifying the network goal, and measuring after a certain time period whether or not this goal has been achieved. This requires that it is possible to quantify the network goals in measurable constructs, which, specifically in interorganizational networks, is not always possible.^22^ Network goals are often defined too vague or not in a measurable way. Moreover, quantified network goals tend to ignore the relational processes behind them.^22^ The second method is asking network participants about their experiences, whether or not they perceive the network as effective. Interestingly, if you ask network members about their ideas to measure the network’s effectiveness, they also indicate to prefer these two possible measures of effectiveness.^23^ Network members appreciate measurable effectiveness outcomes over process outcomes, but without the possibility of quantifying network goals, relying on process measures is often considered as the next best thing.^23,24^ Importantly, network members’ positive perception of the network’s effectiveness keeps them involved, and is important for the network’s sustainability.^12,25^

####  Network Involvement

 One of the factors that is often related to network goal attainment, is member involvement. Network involvement can be defined as the extent to which a network member spends time and effort on network activities such as attending meetings, collaborating in projects or being a board member. Greater network involvement leads to cooperation and sustainability.^26^ Involvement also increases familiarity and trust and achievement of collective goals, which in turn increases the quality of relationships.^27^ Scott and Merton^28^ show that collaborative programs achieved their goals due to hard working members, *despite* the many obstacles and transaction costs related to collaboration across organizational boundaries. Hard working members solved complex problems and they persevered also in enduring times. Their involvement ascertained network stability, which in turn increased network effectiveness. This suggests that, if network involvement *decreases*, this also negatively affects the attainment of network goals. This also suggests that member involvement is not a given and that maintaining a sustainable network relies on the involvement of organizational network members in the network.

 Involvement of network members towards the network goals has been found to affect their perception of the network’s effectiveness.^29^ Network members perceive the network as more valuable when they are more involved in the collaboration, and when the benefits of the collaboration become more apparent. This may lead to their perception of the network goal as better achieved.^30^

 When collaboration becomes hard, but network members work harder together, they feel more positive about achieving the network’s goal.^28^ Involved members will show behavior that is in accordance with the network goals, and exert greater effort may problems arise, resulting in higher perceived network goal attainment. Likewise we expect that *low* network involvement will lead to lower perceived network goal attainment. Yet, there may be pathways where also *high* network involvement leads to *low *perceived network goal attainment. In some cases, when network members are highly involved, they can still become dissatisfied – as the hospital CEO indicated. For example, when network members compare their inputs and outputs against those of other members.

####  Social Comparison

 Lately scholars are increasingly interested in informal or interpersonal factors that drive network effectiveness, such as trust, communication, respect, consensus, and involvement.^31^ Also, motivations, priorities, resources, work practices and expertise of their members are acknowledged to affect network effectiveness.^32,33^ There is an increasing acknowledgement of network actors’ agency: within structures of organizations and institutions, members act autonomously.^34^ However, much of this research is about interpersonal processes, while in this research we aim at interorganizational processes. Until now there is not much research about social comparison processes between interorganizational network members, while we have reason to suspect that this mechanism also plays a role in (perceived) network goal attainment.

 Collaborating in a network is for most organizations not only a beneficial engagement: it is also a costly endeavor. Organizations invest human and financial resources to participate in a network, while the benefits most likely flow into the network instead of back to the single organization.^35^ Therefore, members agree on their expectations of each other, before collaborating, to monitor and adjust their interaction.^36^ These agreements are necessary because organizations want return on their investments – and compare their returns to the investments and returns of other organizations in the network.^37^

 By social comparison, we mean the process of thinking about one or more people in relation to the self.^38^ This process means that people look for similarities or differences between them and others on certain dimensions. Social comparison entails acquiring information, thinking about this information and assessing one’s own performance against the information they received about some other person, and finally reacting to this information. The reason why people engage in social comparison is to obtain information about their relative position as opposed to other’s position on that same dimension. While this information aims merely at interpersonal social comparison, the same phenomenon is omnipresent in organizational life.^39^ Festinger’s initial social comparison theory states that people compare their input and output to that of others. Based on this assessment they decide the level of fairness of the distribution of outcome according to Homan’s theory of distributive justice.^39^ Moreover, Greenberg et al^39^ also state that the “fairness” judgements are based primarily on social comparison rather than on objective information. Prior research on social comparison in organizations, shows that affective commitment (how attached people feel to their organization) as well as job satisfaction decrease when people engage more in social comparison.^40^ Even though the cited study is about coworkers in an organization, there is reason to assume that these findings can be generalized to the reality of interorganizational network settings. As Kenis and Raab^41^ describe, the organization of an interorganizational network is comparable to that of an organization. So this is reason to believe that network members who engage more in social comparison in an interorganizational network show the same mechanisms as coworkers in an organization: they retreat and feel less positive about the organization.

 Organizational network members engage in this comparison especially in a situation where they have to invest resources to collaborate towards a collective goal.^29^ Based on the outcomes of this comparison, organizations change their behavior strategically^42^: they either adapt their expectations of what the network can achieve in general or for their organization, or they adapt their behavior and reduce their involvement with the network.^43^ Hence, social comparison can be a contribution to the configurations of conditions leading to low perceived goal attainment.

 In a nutshell, more involvement with the network entails that return on investment becomes more important for organizations, specifically in relation to other organizations. Therefore high involvement combined with high social comparison will lead to *low* perceived network goal attainment.

####  Organizational Goal Achievement

 Interorganizational networks consist of different organizations who pursue their own organizational goals, next to the network goal.^4,44^ If organizations acknowledge the network goals and strive for them, obtaining the network goal becomes easier, because organizations will show behavior in accordance with the network goal.^44^ However, participants in a network are also loyal to the organization they represent, starting with the fact that network membership is often supposed to benefit the organization.^9,16^ When organizational interests prevail over network interests, network goal attainment may suffer.^45^ This is specifically the case when network participation is costly for member organizations, but the return on their investments is not directly flowing back into their own organization.

 Achieving the network goal is less important to organizations who are in the network mostly for their own benefit.^46^ In this situation, network members pursue organizational goals rather than the network goal, which affects their activities within the network.^47^ For example, network membership may bring privileges or reputation benefits to organizations, without requiring much effort or investment from the members. Perceived network goal attainment in these situations is parallel to achieving their organizational goals. However, network members who are more committed to their own organizational goals are likely less committed to network goals.^33,44^

 To summarize, network membership is expected to be a double edged sword that should help in obtaining the network goals, and while doing so increase sustainability of individual member organizations. Therefore, we expect that high involvement, combined with the pursuit of organizational goals with network membership, contributes to low perceived network goal attainment.

 It is important to note that in the above, it is the perception of network members that drives their evaluation of (the input of) others and the effectiveness of the network. We know that the perceptions of actors of the network itself and the actions of others are often highly inaccurate.^48^ This is primarily a problem for studies that utilize “objective” network structures or positions as it is unlikely to what extent actors actually possess that objective information or use it as a basis for their decision-making. For our arguments, it is actually a strength as research also shows that network actors base their decisions on the information they have even when it is highly inaccurate.^49^ The poor cognition of network members could even be an explanation for why some networks perform poorly^50^ as it gives rise to mismatches in behavior between network members. We will briefly explore this notion in the discussion section of this paper.

 In sum, we propose several different pathways that explain why network members may or may not be satisfied with the achievement of common network goals. First, we suggest that the level of involvement may play a role in perceived network goal attainment. Next, we describe the role of social comparison – the way network members perceive other members’ efforts and input towards the common goal, affects the way the way they perceive the network’s goal attainment. Last, we contend that the member’s involvement interacts with obtaining organizational goals and that this is also a pathway to lower perceived network goal attainment.

## Methods

###  Research Setting

 We test our hypotheses in a local PCN in the Netherlands. general practitioners (GPs) in primary care receive specific funds for the organization and infrastructure of primary care in the region (“O&I funds”) from healthcare insurers. These funds can be used for organizing better quality integrated primary care provision in regional or local networks.^51^

 Dutch healthcare law obligates a basic healthcare insurance for every citizen of the Netherlands. Insured people pay a premium – the maximum amount of this premium is decided by law. Regulations also describe which types of care are covered by insurance and for which types of care an additional fee is required from the patient. Primary care by GPs is covered by basic insurance.^52^ GPs receive a flat fee for every patient that is described to their practice, while other primary care providers depend on the influx of patients for their income.

 PCNs are increasingly prevalent in the provision of care. They are effective in providing better care with good clinical outcomes for many patient groups.^53,54^ In the Netherlands, GPs function as gatekeepers. They make sure patients receive the care they need, but also need to prevent overburdening of specialist care by limiting referrals.

 The PCN we study serves a population of approximately 20 000 inhabitants in two adjacent towns in the eastern part of the country. It is comprised of 29 organizations that provide different types of primary healthcare, and a network bureau that facilitates the network members in achieving the common goal. The aim of this network is to provide high quality, personalized, integrated primary care to the citizens of these two towns. The network is governed by a board occupied by network members, and a bureau that supports network activities. The bureau is staffed by a director, a policy advisor and a secretary. About half of the organizations are located together in one building, just outside the center of one of the towns, while the other half of the organizations are spread across the two towns.

###  Analytical Approach, Methods and Measures

**Figure 1:**
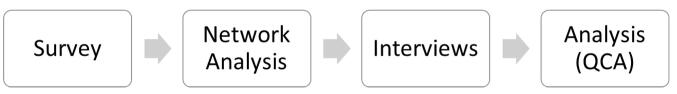
Research Process. Abbreviation: QCA, qualitative comparative analysis.

 We rely on two main data sources (Figure 1). First, all network members and the bureau staff were invited to participate in an online survey in June 2020. Participants were organizational representatives. They were mostly practice owners and the main, and sometimes only, care provider in their organization. Therefore, the conventional issue related to the respondent’s limited knowledge about the subject under investigation—particularly in the case of large organizations—was less of a concern in this context. Prior to participation, respondents were informed about the aim and content of the study, and asked to provide their informed consent.

 Second, a purposive sample of eight representatives from organizational network members was invited for in-depth qualitative semi-structured interviews in July 2021. We invited a sample of board- and non-board members, GPs and non-GPs, and organizations located within and outside the health center to participate in these interviews. Interviews took place within the practices of the respondents. Due to COVID-19 measurements, preventive measures were taken such as physical distancing and proper ventilation; masks were no longer indicated.

 In the survey, participants were first asked to indicate with which network members they collaborate on projects and with whom they communicate about network related tasks and responsibilities. This information was used for the network analysis. We calculated degree centrality scores, and network centralization and density. This informed our choice for the purposive sample for the interviews. Next, the participants were asked to assess network goal attainment, as well as their own commitment in the network and that of their partners. The survey also contained questions regarding obtaining organizational and personal goals.

 The strength of applying this multi-method approach is that we can combine the best of these methods to get a holistic view on the mechanisms we study. Moreover, combining these methods provides stronger support for our findings, despite our sample being small. Findings can be more confidently presented through the support of combining several methods instead of applying only one.

####  Operationalization

 Our dependent variable (DV) was perceived network goal attainment. In line with Peeters’ work^21,23^ we use perceived network goal attainment as a proxy for network effectiveness in general. The network we studied did not have a clear, measurable goal definition nor did they formulate performance or effectiveness indicators. Therefore we argue that the best approach to measure network effectiveness was to ask network members their perception of how well the network currently achieves its goals.

 We measured three independent variables (IV). Involvement was measured by participation in network related tasks and responsibilities in line with Klijn et al.^55^ The accumulated activities accounted for the involvement variable. We also measured involvement by degree centrality of the network members, following Huang and Provan.^56^ This yielded the same results on the variable involvement.

 The variable “social comparison of input” was operationalized as the difference between how respondents asses their own commitment minus how they assess the commitment of other actors in the network following the definition of social comparison by Greenberg et al.^39^

 The variable “organizational (org) goal attainment” was operationalized as the level at which the respondents indicate to achieve organizational goals by network participation.

 Location and organization type are used as control variables. GPs are subject to different rules and regulations than the other providers,^52^ and they are overrepresented in the sample. An overview of the operationalizations of the variables of interest is provided in the appendix (Table 1).

**Table 1:** Operationalizations Table

**DV: perceived network goal attainment**	“How well do you think the network currently reaches its goal?” Likert scale 1-5 (Not at all – A lot)
**IV: network involvement**	Sum of dummy variables: Board member (0 = no board member); Participating in projects (0 = no participation); Attending network meetings (0 = no meeting attendance)
**IV: social comparison**	Difference: Commitment – Commitment of others Commitment: “How committed do you feel to the network?” Likert scale 1-5 (Not committed at all - very much committed) Commitment of others: “How would you assess the commitment of other members within the network?” Likert scale 1-5 (not committed at all – very much committed)
**IV: organizational goal attainment**	Mean of (organizational goal 1+ org goal 2 + org goal 3)/3. “Please indicate to what extent you reach the following goals by participating in the network: Org Goal 1: Increasing the number of my patients Org Goal 2: Increasing the public awareness of my practice Org Goal 3: Increasing the options for diagnosis and treatment of my patients.” Likert scale 1-5 (not at all – a lot)
**Controls**	Location: Dummy (0 = outside the health center) Organization type: dummy (0 = not GP)

Abbreviations: DV, dependent variable; IV, independent variable; GP, general practitioner; org, organizational.

####  Analyses

 We applied a mixed methods design. First, the network was analyzed using social network analysis in UCInet to get a better understanding of each member’s position within the network; in line with work by Mukinda et al.^57^ Following this, interviews were held with a purposive sample (n = 8) based on their network structural position and their characteristics. In the interviews we could elaborate on underlying experiences and explanations related to network involvement, social comparison and organizational- and network goal attainment.

 The interviews were analyzed using thematic analysis. Audio files of the interviews were transcribed verbatim and coded with Atlas.ti by the first author. First, a round of open coding was conducted where the first author derived specific themes. In a second round, the author specifically coded on the themes “comparison,” “investment,” and “returns”; as well as “organizational goals” and “network goals.”

 We applied QCA on the survey results, which has added value as it is specifically developed to analyze small sample sizes. In a nutshell, QCA is a comparative case analysis, where each case is considered as a combination of attributes.^58^ Different combinations of attributes can cohere with the presence of an outcome, in our case low perceived network goal attainment. Importantly, this implies that QCA can deal with settings in which multiple combinations of attributes lead to the achievement of an outcome (ie, equifinality). This is important because our model contains different pathways that could each result in low perceived network effectiveness. Given the space limitations of a multi-method paper we refer to Greckhamer et al^59^ for a more comprehensive discussion of the QCA method.

 As input for the QCA analysis, both the attributes and outcome are transformed into sets. We use the survey data described in the above and calibrate this for a crisp-set QCA. We distinguished organizations with high involvement (score of 3), with high social comparison (score of 0 or higher), with high organizational goal attainment (>3), and with *low* perceived network goal attainment (below average). Using this calibrated data the QCA was performed using R.^60^

## Results

 We start with a description of characteristics of respondents in the sample, followed by a brief description of the network analysis. After that, we present the results from our interviews. Following that, we report and discuss the findings in our QCA.

###  Respondent Characteristics

 The survey resulted in a response of 67% – 20 respondents out of 30 organizational network members. Table 2 provides an overview of the characteristics of respondents in this study. Respondents were either directors or owners of the organizations involved, and mostly also the main care provider in their organization. A nonresponse analysis shows that there was no difference in response rate between organizations located within or outside the health center, as well as no difference in response rate between organization types.

**Table 2:** List of Practices Involved and Their Locations

**Practice Type**	**Total N**	**Located in Center**	**Response**
GPs*	6	6	3
Paramedical (physical**-; occupational-*; practice*- therapy and podiatry)	13	4	10
Psychology*	3	0	3
Elderly care	2	2	2
Speech therapy	2	0	0
Other (apothecary, dietician*, laboratory)	3	3	1
Network bureau*	1	1	1
Total	30	16	20

Abbreviation: GPs, general practitioners. An asterisk (*) indicates the practices that were invited for, and participated in, an interview. There are two asterisks because two physical therapy practices were invited.

 In general, respondents are neutral towards achieving the network goal (M = 3.15, SD = 0.75). All members have at least a minimum level of involvement since there are no respondents in the group “no involvement (0).” Social comparison scores show that in general, members think they are more involved with the network than others do (M = 0.300, SD = 0.801). Network members indicate that network membership is important for their own organization; organizational goal attainment mean is 3.17 (SD = 0.988). Split per location (within, or outside the health center) the scores show similar results. An independent samples *t* test shows that the scores on social comparison and involvement differ significantly for members within or outside the health center. Members within the health center feel more involved (M = 2.55) than outside the health center (M = 1.3; *P*> .001). Members inside the health center also engage more in social comparison (M = 0.727) than the members outside the health center (M = -0.22; *P*< .005) (Table 3).

**Table 3:** General Scores on Variables

	**Location**	**n**	**Mean (SD)**	**Min-Max**
Involvement	In	11	2.55 (0.82)	1–3
	Out	10	1.3 (0.48)	1–3
Social comparison	In	11	0.727 (0.786)	-1–2
	Out	9	-0.22 (0.44)	-1–2
Organizational goal attainment	In	10	3.125 (1.088)	1–5
	Out	9	3.3611 (0.88)	1–5
Perceived network goal attainment	In	11	3.09 (0.831)	2–5
	Out	9	3.22 (0.677)	2–5

Abbreviation: SD, standard deviation.

###  Social Network Analysis

 Based on survey data, we visualized the network of all the practices that are member of the network. The network has a density of 0.385 and a degree centralization of 0.548. The board members rank among the highest with betweenness centrality; one GP practice that delivers the chair of the board has the highest betweenness centrality (5.754). This means that the board is very well connected to other members and that they likely have broker positions. The GPs also have the highest in-degree centrality, with the practice delivering the chair as most central in the indegree network (0.569).

**Figure 2:**
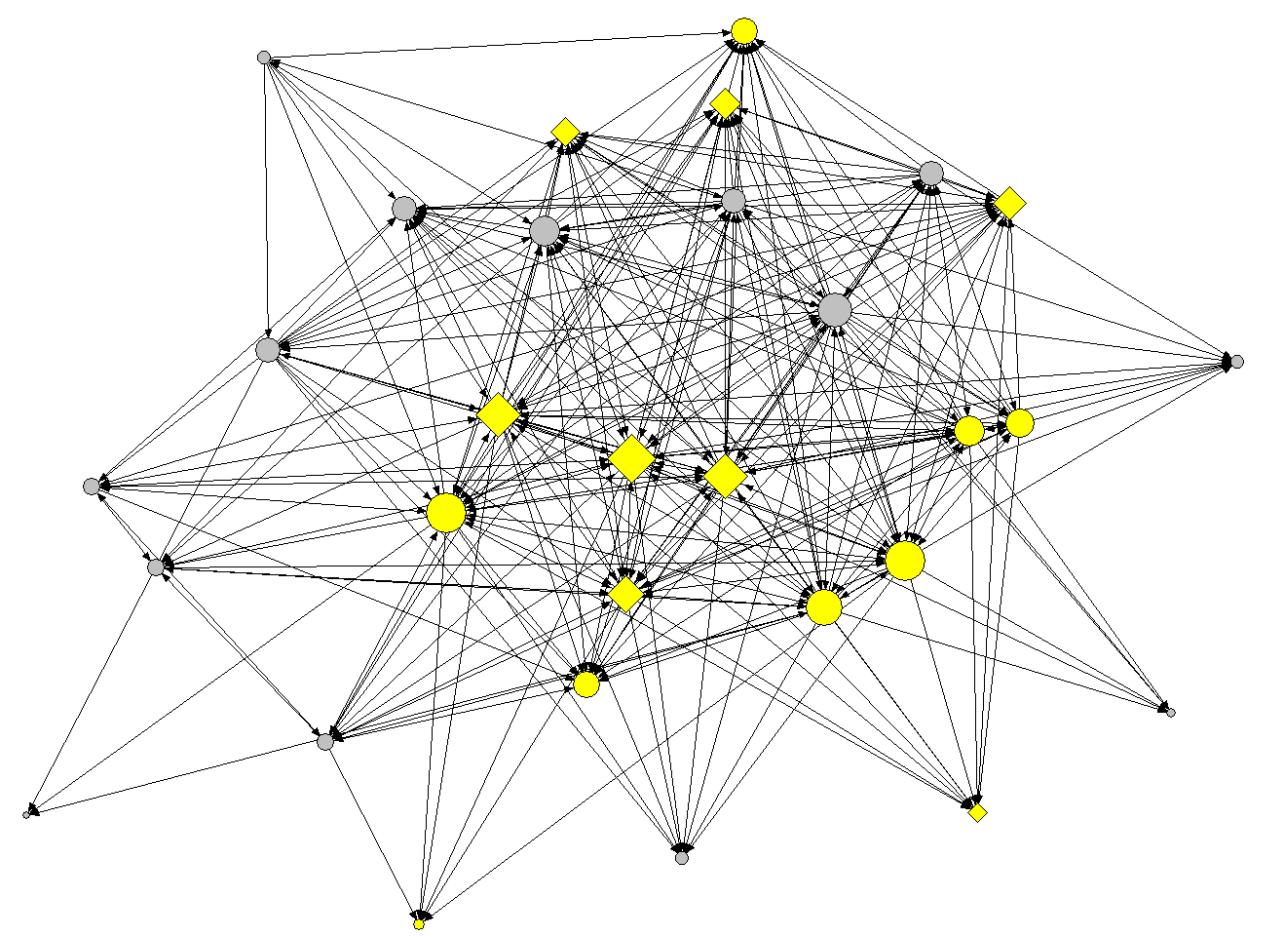
Visualization of the Collaboration Network. Diamonds are board members. Yellow nodes are organizations located within the healthcare center.

 The network structure (Figure 2) shows a strong effect of location – organizations located within the health center tend to be better connected to other members within the health center, specifically to board members.

###  Interviews

 A purposive sample of eight care providers/practice owners, based on involvement and structural network position, was invited for interviews to dive into the themes of social comparison, organizational goal attainment and their perceived network goal attainment. A conversation of one hour gave the opportunity to elaborate on our topics of interest.

 In our analyses, we differentiated between members with low, medium, and high levels of involvement and considered whether they were located within or outside the healthcare center (Tables 4, 5, and 6). Unsurprisingly there were no members with low involvement located within the health center, as there were no members with high involvement located outside the health center.

**Table 4:** Respondent’s Quotes About Network Effectiveness Related to Their Involvement With the Network

	**Low Involvement**	**Medium Involvement**	**High Involvement**
Inside		*“I am not disappointed. You hear me, NOT disappointed. (…). There are periods that it does not work at all, but we also have periods that it goes smoothly. But, for the most part, I think it runs smoothly.”*	*“If you look at the lists, all the projects we run, you can see that we are doing well. Of course, there are always things that can be improved; more patients, more provider involvement… But, I think that with our projects, we truly do something special for our citizens.”*
Outside	*“I don’t really have an opinion about the network’s effectiveness.”*	*“Well, first we wanted to obtain funding for the therapy we provided. That did not work out in this network. Also, the lack of GP’s commitment is a recipe for failure of the network if you ask me.” *	

Abbreviation: GP, general practitioner.

**Table 5:** Respondent’s Quotes About Social Comparison, Related to Their Involvement With the Network

	**Low Involvement**	**Medium Involvement**	**High Involvement**
Inside		*“Look, I am an entrepreneur, so when I see work, I take it. And you know what, I really dislike having to urge people to do something. (…) So maybe, yes, we should activate people more proactively, or increase our expectations.” *	*“The paramedics rely on patients for their income, their food. So if they see some other sells well, and their patient goes there, they can easily say: but I can do that kind of care, too. (..) Every patient counts, that is our reality.” *
Outside	*“I don’t really pay attention to what others do in the network. Should I have an opinion about that? No, I don’t really know.”*	*“I cannot really assess how much the others invest. Hard to say. (…) There are people who never show up for meetings, for example. I don’t really judge that, but it surprises me; how can you be a member of this network and never show up?”*	

**Table 6:** Respondent’s Quotes About Obtaining Their Organizational Goals Related to Their Involvement With the Network

	**Low Involvement**	**Medium Involvement**	**High Involvement**
Inside		*“The patients find me. I get support. So, what I mean is, we are negotiating with the insurance company, the director does that for us so I don’t have to. (…) I just want the best care for the patients.”*	*“I just think about our patients – because ultimately we are here for our patients and we need to include them in our projects. And yes, very often their trajectory starts with us, the GP’s. You just need to think of your patient’s needs.” *
Outside	*“Our collaboration with the network is to provide therapy together with the physical therapist. (…) I don’t really see the need to be more involved than that actually. I don’t depend on them for patients, because there is enough work.”*	*“You also look into what it can provide for you. I mean, how will it benefit me? Is it the content, or the name? (…) It does not make sense to invest so many hours per week while you get nothing in return.”*	

Abbreviation: GP, general practitioner.

 Our interviews show that most network members are quite neutral about obtaining the network goals—they are not very positive, nor very negative—while GPs are more satisfied with the level of network goal attainment.

 Organizations that are more involved with the network tend to compare their own input more to that of others, while organizations with low levels of involvement are less interested in the input of others.

 Organizations that are less involved with the network, are primarily focused on obtaining their organizational goal, while achieving the network goal is less important to them. This also makes them almost indifferent to the functioning of the network as a whole, because the existence of the network is merely to help them obtain their organizational goals. Simultaneously, organizations with high levels of involvement, are primarily concerned with obtaining the network goals and perceive obtaining their organizational goals as less important.

 Besides the role of social comparison and organizational goal attainment, one of the factors that was consistently discussed by all participants, was the influence of payment regulations and market forces for the organizations involved in the network. The care provided by some organizations is paid by basic insurance from the Health Insurance Law, while other care is primarily paid for by the patient itself. This means that those organizations that rely on “direct access” (with no interference by a GP, paid by the “customer”) are network members for different reasons. One of the explanations is that for these organizations, organizational goal achievement may be more important than network goal achievement, because they simply do not have the luxury to think outside their organizational boundaries.

 “*Collaboration appears to be difficult for many people. Let’s not forget we are all also competitors”* (within center, high level of involvement).

 “*The difference is, every citizen needs a GP. We don’t need to entrepreneur. (…) We get paid per patient that subscribes to our practice, while other organizations earn their income from every patient they treat”* (within center, high level of involvement).

 The network director confirmed our most important findings during his interview:

 “*Some practices are very active, while I rarely see some of the others. Well, yeah, they also gain less I think. They are less visible. GPs will refer less to them and they gain less profit from participating in projects – there is always something to gain from that. (…) It does not make me angry, it’s just, I just focus on the group that is reliable and active. (…) For GPs it is different. They do not depend on participating in the network for their profit. (…) The GPs do not have any interest for their organization, it does not render extra patients, or extra interesting cases. They are more idealistically driven. And for the others, they do not invest a lot of time, but they also gain much less. So that is fair, I think” *(network director, within center).

 The insights from the interviews reflect our expected pathways as well. Members who feel less involved with the networks, do not feel a tendency to estimate others’ input or compare it with their own involvement, so this also has little effect on how they perceive the network goal attainment. Once members become more involved, they engage more in social comparison and become disappointed when they see others put in less. The importance of obtaining organizational goals is independent of involvement, but matches with the type of organization: GP’s organizational survival relies less on network membership, while other types of organizations, such as physical therapy practices, rely for their income on a steady influx of patients, hence, for them, organizational goals may be more important. If the network cannot achieve that for them, they can become disappointed as well. However, as the director states: input and output are in general well divided, so investing less time and effort in the network usually means the member gets fewer benefits out of membership as well.

###  Qualitative Comparative Analysis

 The results from the crisp-set QCA in which we assess which combinations of attributes cohere with *low* perceived network goal attainment are largely in line with those of the interviews but add additional nuance (see Table 7). Specifically, we find three pathways to low perceived network goal attainment.

**Table 7:** Qualitative Comparative Analysis Results for Low Perceived Network Goal Attainment

**Causal Conditions**	**1**	**2**	**3**
	**Frontrunning**	**Free Riding**	**Over-Aspiring**
*High involvement*	⬤	⊗	⬤
*High social comparison*	⬤		⊗
*High organizational goal attainment*		⬤	⊗
Coverage	0.20	0.40	0.05
Consistency	1.00	0.75	1.00
Overall solution coverage	0.65		
Overall solution consistency	0.85		

⬤ = condition present; ⊗ = condition absent; blank space = the causal conditions may be present or absent.

 This first pathway, labelled “frontrunning,” captures a process where organizations that are highly involved, rate their own input into the network higher as that of their peers. Regardless of their level of organizational goal attainment, these organizations score low on perceived network goal attainment. One could say that these organizations are so involved that they become disappointed with the input of others and therefore with the results achieved collectively. Upon closer inspection, one organization for elderly care that was the main instigator of this network, and a physiotherapist, are the frontrunners of this network. This mechanism provides support for our second hypothesis.

 The second pathway, labelled “free riding,” captures organizations that are not highly involved but do score high on organizational goal attainment. This is the group with the largest representation. All psychologists, some physical therapy providers and an elderly care organization fall into this category. Regardless of their level of social comparison, these organizations also score low on perceived network goal attainment. These organizations are mostly in the network for their own gains and not for the collective achievement. This pathway nicely overlaps with the our prediction in hypothesis 3.

 Interestingly, we find a third pathway, “over-aspiring” which combines high involvement with low social comparison and low organizational goal attainment. This captures organizations that put a lot of time and energy in the network but still feel others are doing more. This group consists of three board members. They might have high expectations of what the network can do, and over-aspire, resulting in disappointment in the actual achievements of the network.

 In short, we can conclude that perceived network goal attainment is affected by social comparison and by the pursuit for organizational goals: either to sustain their organization or to obtain common goals. Network members may become disappointed in what the network can achieve, when other members are less involved, or when they need the network for survival of their own organization.

## Discussion

 With this empirical research we investigated how involvement affects network members’ perceptions of network goal attainment. Our case study was a local PCN, governed by a board and a network bureau. We applied multiple methods to support our findings: practice owners participated in a survey and a smaller sample participated in interviews. The results were analyzed with social network analysis and QCA.

 We find that highly involved network members compare their own investment to the network to the investments of others, and as a result they become pessimistic or disillusioned towards obtaining the network goal. We also found a contributing effect of organizational goal attainment. When network members are more involved with the network, they also indicate they obtain organizational goals, and become pessimistic towards obtaining the network goal.

 A specific finding, that we did not anticipate, was that different rules and (financial) regulations affect how members perceive the network’s goal attainment. GP practices by definition play a different role in the network, due to their function and secure payment regulations. All other organizations in the network depend on steady patient flows, referrals by the GPs, because they are subject to different payment regulations. They may be more committed to their organizational goals than to the network goals. GPs benefit by collaborating with these practices, because collaboration results in advantages for them: improved information sharing and sharing resources, hence improving quality of care. For the healthcare providers whose care is not paid by insurance, being a member of these networks is beneficial because of a more steady flow of patients via GPs.

 Moreover, all the GPs are located within the same building, which also affects the collaboration within the network. This points toward another finding of our study, namely the importance of geographical—or even physical—closeness. Organizations that were located within the same building tend to be more involved and consequently have more opportunities to become more influential, such as occupying board positions. Sharing the same location may result in in-group favoritism,^61^ which only strengthens the effect of location, and slowly leads to phasing out the organizations located outside the health center. It creates inequality of opportunities for network members: larger organizations that have enough resources to invest in network participation will become more influential, and in the process they become more well-known and generate more revenue for their own organization. This is a specifically interesting finding since this research was executed during the COVID-19 pandemic. Care providers were still providing care but all other network activities, such as member meetings etcetera, were cancelled. In other times these would have strengthened personal contacts, but by lack of personal contacts, the already strong ties from members within the health center could have become stronger.

 Last, we found differing value propositions of network membership. As one respondent put it: “Increasing options for diagnosis and treatment of patients (organizational goal 3) should be the network goal,” which indicates that organizations may have different goals in mind with participating in the network than contributing to the network goal.

 This article contributes to the scarce literature on network effectiveness, that is currently mainly focused on its technical predictors. We uncovered how social comparison and organizational goal attainment mediate the relationship between involvement and perceived goal attainment.

 Next to our theoretical contribution to the knowledge of sociodynamic processes within networks, we also contribute with this research to the current knowledge about measuring network’s effectiveness. While traditional methods to measure effectiveness have proven insufficient for network contexts, measuring network effectiveness by asking members about their perceptions also proves problematic. Network member’s perceptions about the networks’ goal attainment may be affected by their perceptions of other member’s efforts to the common goals or by how well network membership helps them gain organizational benefits. In this light it seems interesting and fruitful to further integrate the research on network cognition with that on network effectiveness. The literature on network cognition has shown that mismatches in perception of the network can result in poor performance^50^ but this literature has primarily focused on cognition of the network structure (ie, who is connected with whom) and less on differences in the cognition of the goals and effectiveness of the network.

###  Limitations and Suggestions for Further Research

 To the best of our knowledge, this is the first study to capture sociodynamic mechanisms and processes at play in interorganizational networks. However, the study took place in a small network in a region in the Netherlands, resulting in a small sample. By focusing on one network, the implications of organizations being involved in multiple healthcare networks were not explored. This could be an interesting research opportunity given the fact that many organizations in healthcare engage in multiple networks simultaneously. This provides challenges to network involvement, because organizational resources such as time and money are limited and can only be spend once. More research in other settings is also needed in order to generalize our findings to broader contexts, specifically with larger networks.

 This research was conducted during the COVID-19 pandemic. Most of the communication with network members took place online. The interviews were planned in a period with only mild COVID-19 regulations, so interviews could all take place live. In our interviews we asked respondents whether they experienced differences in the collaboration between network members. The respondents indicated unanimously that COVID-19 did not affect collaboration ties, besides most meetings taking place online instead of live. However, opposite to what we expected, the effect of location was further established during the period of working online: collaborations within the healthcare center, were easier to sustain than collaborations that were weaker or not yet well-established.

## Conclusion

 Current knowledge about determinants of network success mostly focuses on technical, measurable aspects related to the organization and management of collaboration. Research on social processes in network collaboration, that affect network goal attainment, is still scarce. With this research we show that, even though organization and management practices may be well organized, there is still a black box of psychosocial processes affecting the interactions between network members.

 We have uncovered important mechanisms that affect network members’ assessment of network goal attainment. When members assess the commitment of others as lower than their own commitment, while their own involvement is high, these members are more pessimistic about network goal attainment. Organizations within a network that strive to obtain their organizational goals may also be more pessimistic about network goal attainment.

 PCNs are increasingly set up as a way to organize healthcare delivery as well as reduce fragmentation, and even though many positive results are expected, they do not always achieve their goals. Healthcare professionals, network managers and mandating organizations such as government or health insurance company, should take into account the mediating role of the sociodynamic phenomena that we studied.

## Acknowledgements

 We are grateful for the help and support of the network’s members in making time to participate in this research project. We also thank the anonymous reviewers for their valuable comments on previous versions of this manuscript.

## Ethical issues

 This research focuses on organizational perspectives. No data from, about or involving patients was acquired so the necessity for ethical approval was waivered. Respondents to interviews and surveys provided informed consent. Agreements were made about storage and safety of the data.

## Competing interests

 Authors declare that they have no competing interests.

## Data availability statement

 Given the sensitive nature and anonymity of the respondents, data is only available upon request with the corresponding author.
